# Physical violence during pregnancy and pregnancy outcomes in Ghana

**DOI:** 10.1186/1471-2393-14-71

**Published:** 2014-02-15

**Authors:** Michelle Sharon Pool, Easmon Otupiri, Ellis Owusu-Dabo, Ank de Jonge, Charles Agyemang

**Affiliations:** 1 Department of Public Health, Academic Medical Centre, University of Amsterdam, Meibergdreef 9, 1105 AZ Amsterdam, The Netherlands; 2 Department of Community Health, Kwame Nkrumah University of Science and Technology, Kumasi, Ghana; 3 Midwifery Science, AVAG and the EMGO, Institute for Health and Care Research, VU University Medical Center, Amsterdam, The Netherlands

**Keywords:** Domestic violence, Intimate partner violence, Early pregnancy loss, Perinatal mortality, Neonatal mortality

## Abstract

**Background:**

In pregnancy, violence can have serious health consequences that could affect both mother and child. In Ghana there are limited data on this subject. We sought to assess the relationship between physical violence during pregnancy and pregnancy outcomes (early pregnancy loss, perinatal mortality and neonatal mortality) in Ghana.

**Method:**

The 2008 Ghana Demographic and Health Survey data were used. For the domestic violence module, 2563 women were approached of whom 2442 women completed the module. After excluding missing values and applying the weight factor, 1745 women remained. Logistic regression analysis was performed to assess the relationship between physical violence in pregnancy and adverse pregnancy outcomes with adjustments for potential confounders.

**Results:**

About five percent of the women experienced violence during their pregnancy. Physical violence in pregnancy was positively associated with perinatal mortality and neonatal mortality, but not with early pregnancy loss. The differences remained largely unchanged after adjustment for age, parity, education level, wealth status, marital status and place of residence: adjusted odds ratios were 2.32; 95% CI: 1.34-4.01 for perinatal mortality, 1.86; 95% CI: 1.05-3.30 for neonatal mortality and 1.16; 95% CI: 0.60-2.24 for early pregnancy loss.

**Conclusion:**

Our findings suggest that violence during pregnancy is related to adverse pregnancy outcomes in Ghana. Major efforts are needed to tackle violence during pregnancy. This can be achieved through measures that are directed towards the right target groups. Measures should include education, empowerment and improving socio-economic status of women.

## Background

The former United Nations Secretary-General, Kofi Annan, in 1999 said “Violence against women is perhaps the most shameful human rights violation and it is perhaps the most pervasive, it knows no boundaries of geography, culture or wealth. As long as it continues, we cannot claim to be making real progress towards equality, development and peace” [1]. Indeed, violence against women is a global problem. It is present in every country, regardless of culture, ethnicity, and socio-economic status [2].

According to the World Health Organization’s study on Women’s Health and Domestic Violence against Women, the percentage of all women from the age of 15 who experienced physical or sexual violence or both, by partner or non-partners, was between 18.5% and 75.8% [3]. In most settings, the intimate partner was most likely to be the perpetrator. Non-partner violence prevalence ranges from 5.1% to 64.6% and violence by an intimate partner from 15.4-70.9% depending on the country [3].

Violence against pregnant women is also an important problem. Literature shows a prevalence range from 1.2% to 51% [4-13]. The wide range in frequency is most likely a reflection of differences in study designs, definitions and study populations [14]. A study in Saudi Arabia showed a prevalence of physical violence during pregnancy of 21% [12]. The husband was the perpetrator in the majority of cases (87%).

Physical violence against women is present in every country regardless of socioeconomic development. Nevertheless, it is more common in certain subgroups in the population than in others. Several factors have been shown to be associated with domestic violence including young age [3,7,15], lower education level [3,7], not being married [7] and being single [15], smoking during (third trimester of) pregnancy [7,15], alcohol and drug use [7], stressful life events and depression [9], as well as lack of faith in God or a higher power and lack of contraceptive use [9]. Additionally, poor quality of the relationship with the intimate partner [16,17], the presence of abuse before pregnancy [15] and fertility factors are important.

There are also cultural, political, legal and economical etiological factors for domestic violence against women. A political factor, for example, is the unequal power between men and women in some societies. Women are underrepresented in politics and in the legal and medical professions. A lot of women are financially dependent on their husbands, and are not employed. These factors contribute to unequal power between men and women. This unequal situation makes women more vulnerable to domestic violence [2].

Many health consequences are related to physical violence: injury, gynecological problems, miscarriage and self-harming behaviors such as smoking [2]. In pregnancy, violence can affect both the mother and the unborn baby. Maternal and perinatal complications can include depression, anxiety, drug abuse and alcoholism [2]. There is also a bigger risk of adverse pregnancy outcomes [17] such as perinatal death [4,7,11] or preterm delivery [7,15,16]. Women who experience physical violence during their pregnancies are more likely than those without physical violence to deliver by caesarean section [8,12]. They are also more likely to be hospitalized for maternal complications [8,12]. These complications may include placental abruption and trauma due to blows or kicks in the abdomen [12].

Although some studies show a clear association between violence during pregnancy and poor maternal and perinatal health outcomes, others did not find a significant association between maternal experience of violence and adverse health outcomes. Ahmed *et al.’s* study in India (2006), for example, found no significant association between violence during pregnancy and post-neonatal and early childhood mortality [4]. Another study in the US found that there was no physical injury following trauma although women still experienced fetal loss [18]. These findings suggest that physical injury or trauma may be less important as a cause of fetal loss.

Reducing child mortality is one of the Millennium Development Goals [19]. To reach this goal it is important to identify the causes of child mortality. Reducing physical violence against pregnant women can, perhaps, contribute to reducing adverse pregnancy outcomes.

In Ghana, physical violence against women is not uncommon. About 36.6% of women aged 15-49 had experienced physical violence at some time; 5.2% had experienced violence during pregnancy [20]. Information on the relationship between violence during pregnancy and pregnancy health outcomes is limited in Ghana [21]. It is therefore unclear whether violence in pregnancy is related to poor pregnancy health outcomes in Ghana. The aim of this research was therefore to study the relationship between physical violence in pregnancy and adverse pregnancy outcomes. We hypothesized that there is a positive relationship between physical violence in pregnancy and adverse pregnancy outcomes in Ghana.

## Methods

### Study area

Ghana is a country situated in West Africa. It has a total area of around 238,540 square kilometers [22]. According to the 2010 Ghana Population and Housing Census the total population was about 24,658,823 [23]. In 2009, the life expectancy at birth was 57 years for men and 64 years for women [24].

### Sampling design

The data used in this research were collected during the 2008 Ghana Demographic and Health Survey (GDHS). The Monitoring and Evaluation to Assess and Use Results Demographic and Health Surveys (MEASURE DHS) project promotes global understanding of health and population tendencies in over 85 low and middle-income countries. It does so by providing technical assistance for the implementation of surveys [25].

The DHS collects data on different subjects such as fertility, child health and nutrition [20]. For this research data on violence during pregnancy (as part of the domestic violence module), child mortality, maternal health and demographic factors were used.

Data were collected cross-sectionally through interviews. Around 12,000 households throughout the country were included in the research. Half of these households (6,141) were selected for the individual women and men questionnaire. Selection of these households as well as the individuals is described in detail in Ghana Demographic and Health Survey 2009, p. 5 [20]. Data were collected through interviews in a three-month period (September to November 2008) in selected households [20].

### Study participants

**Figure 1:**
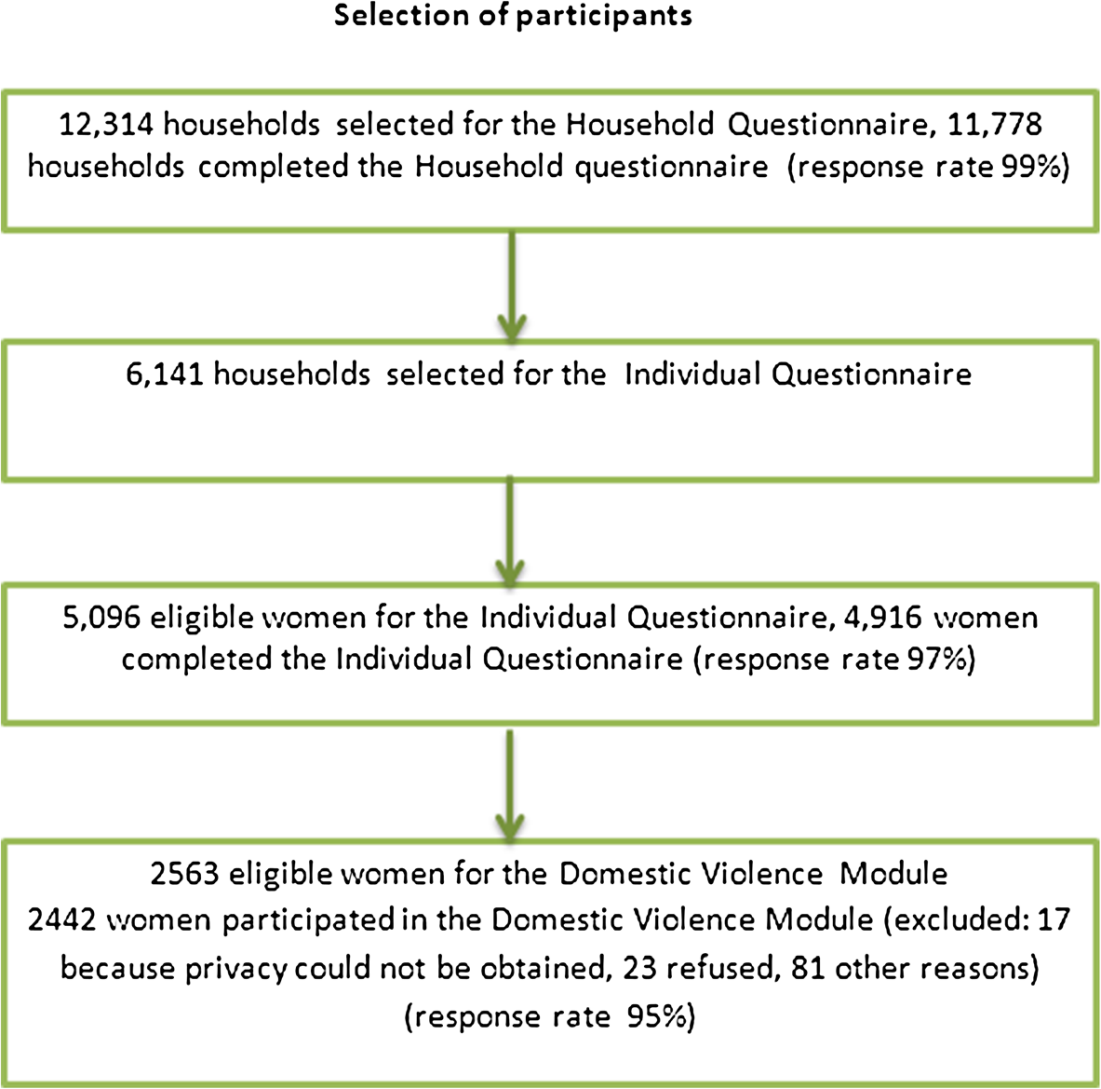
Selection of participants.

For this research the individual women’s questionnaire was used. All eligible women between the ages of 15 and 49 in a half of the households selected for the household questionnaire were selected for the individual women’s questionnaire. In this module the response rate was 97% (4,916 of 5,096 eligible women). The main reason for non-response was the absence of an eligible study participant in a selected household [20]. From the women selected for the individual interview, 2563 eligible women were selected for the domestic violence module. Of these, 17 were excluded because privacy could not be obtained, 23 were excluded because they refused and 81 were excluded for different reasons. From the 2563 eligible women, 2442 completed the domestic violence module. After exclusion of missing values and adding the weight factor for domestic violence respondents, data for 1745 respondent were analyzed (Figure 1) [20].

### Measurements

Domestic violence is a difficult subject to collect reliable data on because; a) the definition of violence differs across cultures and individuals; b) domestic violence is a very sensitive topic and often people do not speak about it, and this can affect the data collected. There were a lot of ethical questions committed to this subject. In order to collect the violence data interviewers were provided training. Additionally, they were provided with a list of contacts for the Domestic Violence and Victim Support Unit (DOVVSU) of the Ghana Police Service and instructed to tell respondents about the Probation and Social Welfare Matters at the district level, where the respondents can go for help [20].

For violence during pregnancy, women were asked whether they had ever experienced violence (i.e. *has anyone ever hit, slapped, kicked or done anything else to hurt you physically?*) during pregnancy, and if so, who the perpetrator was. Additionally, women were asked whether they had ever had a pregnancy loss as a consequence of the (last) husband’s/partner’s actions. However, this does not necessarily include women who were pregnant for a certain time period (i.e. at least 7 months) (see definitions of adverse pregnancy outcomes below) [20].

Child mortality is also a challenging subject to collect data on. Misreporting and omission are not uncommon [20]. Although reliable data on child mortality are still difficult to collect, retrospective surveys such as the 2008 GDHS are more accurate and representative compared to vital registration systems [20]. For terminated pregnancies data collection problems were conceivable due to the sensitivity of the subject. Respondents may find it difficult to talk about terminated pregnancies and thus fail to give accurate information.

To collect information on child mortality, the birth history section of the questionnaire for women was used. A couple of questions were posed to women of reproductive age (15-49), including information about their sons and daughters such as the place they live and whether they are alive or not. For every child that died, the age at time of death was reported. In case of a terminated pregnancy, the month when the last such pregnancy ended was reported. For research outcomes the following definitions were used; early pregnancy loss was defined as loss of the last pregnancy before 7 months gestation. Still birth was defined as loss of the last pregnancy after at least 7 months gestation [20]. Perinatal mortality was calculated as the sum of stillbirth and early neonatal mortality [20]. Early neonatal mortality is mortality of all children between birth and the first 7 days of life [20]; and neonatal mortality was defined as the probability of dying within the first month after birth [20].

In addition, data on demographic and socio-economic characteristics were collected through a questionnaire [20]. Total children ever born were re-categorized into 4 or fewer children and 5 or more children. Education was categorized into no education, primary and secondary and above. The wealth index was based on household income and expenditure. It included information on possessions and characteristics of the household. Marital status was categorized into married or living together, and not living together, widowed or divorced.

### Ethical considerations

Ethical protections built into the questionnaire were in accordance with the World Health Organization’s recommendations for research on domestic violence [26]. The interview could be completed only when strict conditions could be met. Only one person per household, randomly selected by a simple selection procedure, could take part in the module. The aim of this was to assure privacy. If privacy could not be obtained, the domestic violence module was skipped. This was also the case if a translator was needed [20]. Informed consent was obtained at the beginning of the individual interview. At the beginning of the domestic violence module additional information was given. Interviewers were instructed to tell the respondent where to go for help. Due to the sensitivity of both child mortality and terminated pregnancies, extra efforts were made to ensure and protect confidentiality. Losing a child or experiencing a terminated pregnancy is a major and grievous event, which may lead to a lot of emotions. People may feel uncomfortable about this subject and therefore conceal child mortality. As described above, interviewers were provided training, as well as a specific training to implement the domestic violence module in an ethical matter.

### Statistical analysis

SPSS 17 (SPSS Inc Chicago, USA) for Windows was used to analyze the data. Frequency tables and cross tables were used to assess prevalence differences. Chi-square tests were performed to assess differences in categorical variables. Two models of multivariable logistic regression were performed to assess the relationship between physical violence in pregnancy and adverse pregnancy outcomes with adjustments for potential confounders. Model 1 adjusted for age and parity while model 2 adjusted for age, parity, educational level, wealth status, marital status and type of place of residence. The results are shown as adjusted odds ratios with corresponding 95% confidence intervals (95% CI). P-values ≤0.05 were considered statistically significant.

## Results

Table 1 shows the characteristics of the study population by physical violence during pregnancy status. Women who experienced physical violence during pregnancy were wealthier and had a higher parity than those who did not experience physical violence during pregnancy. There were no differences in age, education level, marital status and type of place of residence between those who experienced physical violence during pregnancy and those who did not.

**Table 1:** Characteristics of the study population

	**No physical violence during pregnancy (n=1654)**		**Physical violence during pregnancy (n=91)**		
	**N**	**Percentage**	**N**	**Percentage**	**p-values**
Age groups					
*15-24*	333	20.1	19	20.9	0.96
*25-34*	604	36.5	32	35.2	
*35-49*	718	43.4	40	44.0	
Parity					
≤*4*	1252	75.7	60	65.9	0.04
≥*5*	402	24.3	31	34.1	
Education level*					
*No education*	446	27.0	23	25.3	0.16
*Primary*	367	22.2	28	30.8	
*Secondary and above*	841	50.8	40	44.0	
Wealth index					
*Poorer*	560	33.8	20	22.2	0.01
*Middle*	540	32.6	42	46.7	
*Richer*	555	33.5	28	31.1	
Current marital status					
*Not together*	353	21.3	22	24.2	0.522
*Together*	1301	78.8	69	75.8	
Type of place of residence					
*Urban*	719	43.5	42	46.2	0.620
*Rural*	935	56.5	49	53.8	

*One missing value.

**Figure 2:**
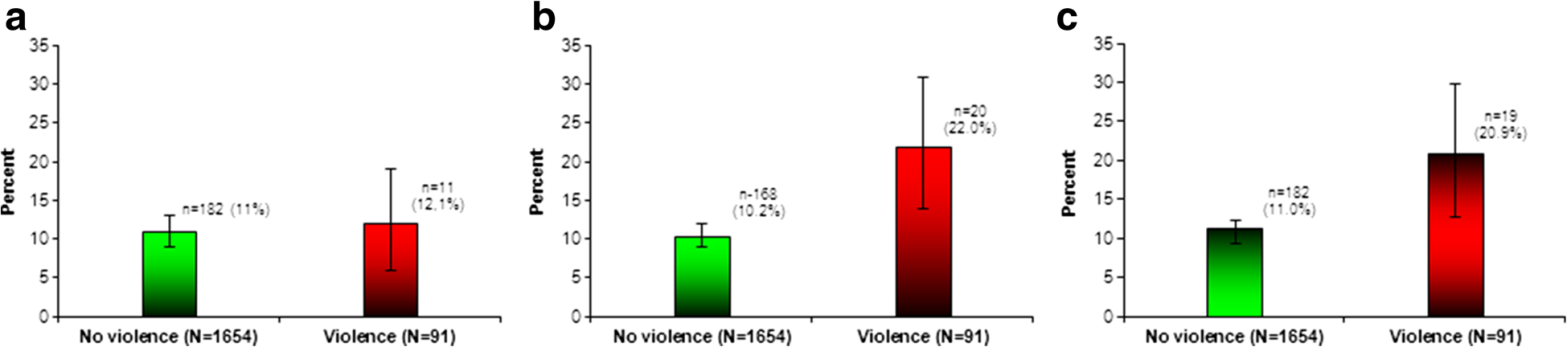
Early child loss (a), perinatal mortality (b) and neonatal mortality (c) by violence during pregnancy.

**Figure 3:**
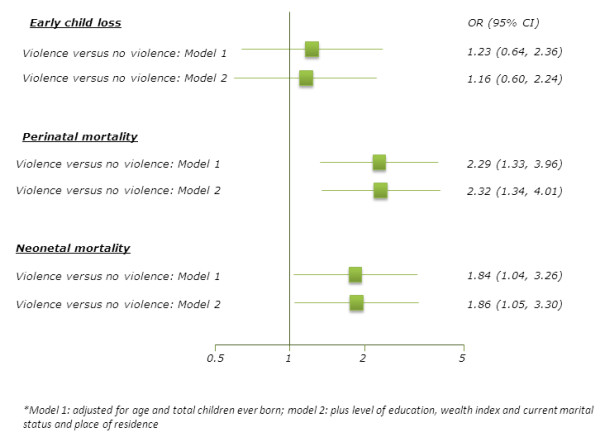
**Adverse pregnancy outcomes and physical violence during pregnancy.** Model 1: adjusted for age and total children ever born; model 2: plus level of education, wealth index and current marital status and place of residence.

Figure 2a-c show the prevalence of early pregnancy loss, perinatal mortality and neonatal mortality by the experience of physical violence during pregnancy. Women who experienced physical violence during pregnancy were more likely than those who did not experience physical violence during pregnancy to have a perinatal mortality and neonatal mortality. There was no difference in early pregnancy loss between those who experienced and those who did not experience physical violence during pregnancy. In the age and parity adjusted models (model 1), physical violence during pregnancy was associated with perinatal mortality (OR = 2.29; 95% CI: 1.33-3.96), and neonatal mortality (OR = 1.84; 95% CI: 1.04-3.26) (Figure 3). After further adjustments for education level, wealth status, marital status and place of residence (model 2), physical violence during pregnancy was still associated with perinatal mortality (OR = 2.32; 95% CI: 1.34-4.01) and neonatal mortality (OR = 1.86; 95% CI: 1.05-3.30), but not for early pregnancy loss (OR = 1.16; 95% CI: 0.60-2.24).

## Discussion

### Key findings

The findings of this study suggest that physical violence during pregnancy is related to perinatal mortality and neonatal mortality, but not to early pregnancy loss.

### Discussion of the key findings

Studies on the relationship between violence during pregnancy and adverse pregnancy outcomes have shown inconsistent results. Studies have been performed in the United States [6-9,15,17,18,27,28], Canada [11], New Zealand [29], Mexico [13], India [4,15], Saudi Arabia [12], Cameroon [5], Kenya [10] and Bangladesh [30]. Although most of the literature on this topic shows associations between violence and adverse pregnancy outcomes [4-8,10-12,14,16-18,29,30], in a research performed by Poole et al. [18], in 5 of the 8 women with fetal losses there was no apparent physical injury. The study was based on retrospective reviews of the medical records of pregnant women diagnosed with traumatic injuries, not specially focused on interpersonal violence. Also, absence of visible physical injury does not guarantee the absence of an associated harmful situation. Therefore that study is not comparable with this current research. Ahmed et al. [4] found a significant relationship between violence during pregnancy and perinatal mortality, however, no significance was found for violence during pregnancy and post-neonatal and early childhood mortality. In Ahmed et al. [4], information from men was used to identify women who experienced domestic violence. This could be a possible explanation for the results obtained. Men may be less likely to report violence and the consequences of the violence that they perpetrated than women who are the victims. Also, men may be less involved in the children’s health than women, this could result in misreporting.

Earlier research in Africa and Bangladesh by Emenike et al. [10], Alio et al. [5], and Silverman [30] using similar methods as our current research found an association between adverse reproductive outcomes respectively for infant mortality and violence and for foetal mortality and terminated pregnancies. Fanslow [29] also found an association between violence during pregnancy and spontaneous abortion and termination of pregnancy. The lack of association between early pregnancy loss and violence during pregnancy in the current study are not consistent with these earlier studies. A possible explanation could be under-reporting in early pregnancy. Women might be more likely to report pregnancy loss later in pregnancy than early pregnancy loss.

The clear relationship between violence during pregnancy and adverse pregnancy outcomes underscores the need to tackle violence against pregnant women. This might include changing the current attitude towards violence. In 2005, the World Health Organization asked women whether they thought men are allowed to use violence in certain circumstances, such as if they did not complete housework correctly, refused sex or were not faithful. Six percent of women from Eastern Europe, and 68% from Bangladesh, Ethiopia, Peru, Samoa, Thailand and the United Republic of Tanzania thought men had the right to use violence in one or more of these circumstances [3]. The situation in Ghana was examined by Amoakohene in his study in 2004 [31] in which 50 women in Accra and Kumasi, two major cities in Ghana, were interviewed. Traditions defined the roles for many women that took part in the research [31]. The expectations of how a woman should act and the belief according to traditional gender norms and role definitions place them in an inferior position in respect to men and give men a sense of supremacy [31]. Although the women interviewed were educated, employed, married and economically independent of their partners, 70% of women interviewed reported the experience of one form of abuse or another [31]. Most women blamed their husbands for the abuse, a minority blamed themselves [31]. However, none of the women who reported abuse went to organizations for help [31]. Reasons mentioned by the women were that: they did not want people to know, that it was justified by their ethnic group and to prevent ridicule [31]. Especially regarding sexual violence there was an atmosphere of silence. Women did not want to talk about this subject. In Ghana, male dominance is a major aspect of society, refusing sex would be a taboo [31]. The attitude of women towards violence has an effect on reporting as well as fighting violence. If women think of assault by husbands as ‘normal’ they may not report it [31], women may not feel the need for change. Also, in case they do report it, acceptance might make it difficult to change. However, acceptance does not mean that there is no need for interventions. On the contrary, women should be educated about their rights, and that culture and tradition do not justify acceptance of violence.

In Ghana, interventions against violence towards women are taken by governmental as well as non-governmental organizations (NGO’s). These organizations operate mainly through education, raising awareness, legislation and investigation, counseling and prosecution of offenders [31]. Chapter five of the Ghana constitution contains fundamental human rights, and in addition specific rights of women. Also, the constitution prohibits all cultural and traditional practices that can injure people [31]. Measures against violence towards women taken by the Ghana government include the passage of the Domestic Violence Bill in February 2007, which empowers the Ministry of Women and Children’s Affairs (MOWAC) to undertake actions against domestic violence such as rescue and rehabilitation of victims [32]. Further actions of the MOWAC include develop standards and performing research [32]. Another governmental institute involved in this subject is the Domestic Violence and Victim Support Unit (DOVVSU) of the Ghana Police Service [28]. The DOVVSU deals with prevention as well as with the victims of abuse. Prevention occurs by educational talks at churches and schools. Victims of violence can visit a DOVVSU office. The DOVVSU staff will listen to them and help them to get the right legal, medical and mental help [28]. However, despite these interventions that are taken by different organizations, the WAJU (now renamed the DOVVSU) showed an increase of incidence of violence against women in 2002 [31,33]. This underlines the need for further research towards measures to fight violence against women. This research can contribute by showing the prevalence and consequences of violence against women in pregnancy.

### Limitations and strengths

For this research cross-sectional data were used. A limitation of cross-sectional data collection is that the causal relationship remains unclear [10]. It is not known at which point during pregnancy or during which pregnancy the violence was admitted. For different reasons some women were not interviewed. In earlier research, women who refused to be interviewed were found to be more likely to have experienced adverse pregnancy outcomes [27]. This could affect the results of this research because of underreporting. Another limitation of this research is over- and under-reporting due to the relatively sensitive topic of violence. Due to many ethical and social issues regarding domestic violence, misreporting is conceivable. Different causes for misreporting are mentioned in earlier research, for example, women who experience violence may be more likely to report adverse pregnancy outcomes, or women who have an adverse pregnancy outcome may be more likely to report violence [7]. Another shortcoming is the lack of information about the violence, such as the severity and the time in pregnancy when it occurred [12], which could provide important information for effective preventive measures.

Although this research shows a significant association between violence during pregnancy and adverse pregnancy outcomes, it is important to take notice of other factors that could influence pregnancy outcomes such as substance abuse, alcoholism and smoking as well as other inherent obstetrics and gynecological condition prevailing. Unfortunately data on these topics were not available for the same period as the domestic violence data. Therefore, adjusting for these factors was not possible.

The data used for this research was secondary data. There are advantages and disadvantages when secondary data is used. Advantages include large sampling and high quality of data. Disadvantages could include lack of accuracy of data and data mismatch. However, the data collection was performed in an accurate and professional setting. Although reliable data on perinatal mortality are still difficult to collect, retrospective surveys such as the 2008 GDHS are more accurate and representative than the vital registration system [20]. Because data were collected throughout the country in a large number of households, it gives information that is generalizable for Ghana and maybe even for other parts of (sub-Saharan) Africa. However, different cultures have different ‘norms’ towards violence against women. Ghana is a multi-ethnic society with different cultural traditions [20]. This should be taken into account for the interpretation of the results. Data collection on domestic violence was in accordance with recommendations of the WHO ethical standard on research on domestic violence [20]. Interviewers were provided with training in order to collect the data in a safe way [20].

## Conclusion

The findings of this study suggest that violence towards women is associated with serious health consequences for pregnancy outcomes and thus underscores the need for measures to fight violence against (pregnant) women. Measures could include empowerment, upgrading the socioeconomic status of women and education of cultural and traditional aspects with regard to human rights [16]. Organizations which offer help to women should be more recognized and supported, so that victims know where to go for help. Finally, an important obstacle is the silence surrounding the subject, in order to take measures to abate violence it is necessary to break this silence.

## Competing interests

The authors declare that they have competing interests.

## Authors’ contributions

MSP and CA conceived the idea. MSP carried out the statistical analysis and writing with major help of CA. EO as well as EOD assisted in writing the manuscript and were of major help with local issues in Ghana. AJ finally, helped to draft the manuscript. All authors read and approved the final manuscript.

## Pre-publication history

The pre-publication history for this paper can be accessed here:

http://www.biomedcentral.com/1471-2393/14/71/prepub
